# The limits of metacognitive control during perceptual decision-making: opting out without improving accuracy

**DOI:** 10.3389/fpsyg.2025.1551665

**Published:** 2025-05-20

**Authors:** Rawa Al Dowaji, Ji Xu, Yimeng Jin, Antoine Porte, Johan Lauwereyns

**Affiliations:** ^1^Graduate School of Systems Life Sciences, Kyushu University, Fukuoka, Japan; ^2^École Nationale Supérieure de Cognitique, Bordeaux Institute of Technology, Bordeaux, France; ^3^School of Interdisciplinary Science and Innovation, Kyushu University, Fukuoka, Japan; ^4^Faculty of Arts and Science, Kyushu University, Fukuoka, Japan

**Keywords:** perceptual decision-making, metacognitive control, opting out, time pressure, task difficulty, risk

## Abstract

The purpose of this study was to examine how the inclusion of an opt-out option affects the metacognitive control of perceptual decision-making under challenging conditions. In Experiments 1 and 2, participants were required to compare the flicker frequency of simultaneously presented stimuli. In Experiment 3, participants had to identify the dominant color in a patch of red and green dots. We hypothesized that, with an option to skip, participants would strategically opt out of trials in which they were uncertain, thus reducing their error rates and improving their overall performance. In Experiments 1 and 2, we compared conditions under time pressure with versus without a skip option. We also varied the risk, or penalty associated with error. By raising the risk, we found that participants tended more often to opt out of the decision. However, this escape behavior did not enable them to achieve better performance. The opt-out decision appeared to impose a cognitive burden, requiring additional effort without yielding a clear advantage. In Experiment 3, we varied the time pressure with a short versus long deadline. We also manipulated the task difficulty with color dot ratios that were easy or hard to discriminate. Participants tended to skip more often in hard trials than in easy trials, whereas the short versus long deadline did not affect the skip rate. Again, the increase in opting out did not lead to reduced error rates. Across the three experiments, we found that factors such as risk and task difficulty elicited escape behavior in perceptual decision-making, without improving accuracy. Thus, the participants demonstrated they could monitor their performance but were unable to achieve strategic metacognitive control with the opt-out option.

## Introduction

Decision-making is a complex process influenced by various factors, including the prospect of gain, task difficulty, and the potential for risk or punishment. In the present paper, we focus on how participants may be able to exert metacognitive control on perceptual decision-making (Vargas and Lauwereyns, 2021). This study aimed to investigate the impact of including an opt-out option on the ability to improve decision accuracy under demanding conditions. In the first two experiments, participants were tasked with comparing the flicker frequencies of simultaneously presented stimuli. In the third experiment, participants were instructed to determine the dominant color within a patch composed of red and green dots. For demanding conditions, we focused on time constraints, risk, and the discriminability of perceptual features.

People often make decisions under time constraints, with deadlines imposing pressure to choose (Attwood et al., 2004; Maboudi et al., 2023). While refraining from making a decision may be costly, it is crucial to be mindful of time and make informed choices within appropriate timeframes (Balcı et al., 2011; Tajima et al., 2019). Imposing time limits on decision-making can significantly influence the decisions made (Kozma et al., 2007; Miletic and van Maanen, 2019; Reddi and Carpenter, 2000). Individuals facing higher timing uncertainty tend to respond more slowly than optimally, prioritizing accuracy over reward rate (RR) in free-response two-choice tasks (Bogacz et al., 2006). Short response deadlines can negatively impact both response accuracy and time in decision-making tasks (Busemeyer, 1985), and modern decision-making models emphasize the intricate relationship between response time and accuracy (Brown and Heathcote, 2008). These models propose that people accumulate evidence for each alternative choice until a decision threshold is reached, at which point they make their choice and initiate a response (Parés-Pujolràs et al., 2021). However, when faced with time pressure, individuals may make decisions at lower thresholds, leading to less evidence accumulation and consequently lower accuracy (Boehm et al., 2014; Malhotra et al., 2017, 2018). Research has consistently demonstrated a systematic relationship between accuracy and reaction time, known as the “speed-accuracy trade-off,” highlighting their interdependence (Garrett, 1922; Heitz, 2014; Karsilar et al., 2014).

Time pressure is not the only pressure that affects decision-making, but also the task difficulty, for instance, as a function of stimulus discriminability. People’s ability to deal with difficult tasks varies according to their cognitive flexibility (Chin and Hayes, 2017; George and Dane, 2016; Westhoff et al., 2024). There may also be important interindividual differences in response biases as a function of prior experience (Lauwereyns, 2010). In classical ethology, escape behavior is considered a reflexive response when specific triggers occur. In neuroscience, escape behavior serves as a valuable model for examining various cognitive processes, including decision-making and action selection (Evans et al., 2019). Avoidance behavior has been a focal point of behavioral and cognitive psychology due to its significant impact on decision-making, and its association with various mental health conditions, including anxiety and obsessive-compulsive disorder (Beckers and Craske, 2017). Recent studies highlighted the complex relationship between fear, confidence, and avoidance behavior.

From another point of view, potential risk is considered a main factor in decision-making (Man et al., 2024; Lauffs et al., 2020; Platt and Huettel, 2008). Individuals often opt to avoid decisions when faced with dangerous or risky situations, particularly when the choice involves two options, one of them leading to large benefits with the possibility of big disadvantages, while the second option achieves fewer positive outcomes with more certainty (Mellers et al., 2019; Slovic, 1987). Risky decisions can be also represented by choosing between a small certain reward immediately or a large reward in the future (Lejuez et al., 2003). On the other hand, high-cost options in terms of punishment represent an example of decisions under risk. Previous reports focused on the role of punishment in intensifying the perceived riskiness of a situation (Simon et al., 2009). One study concluded that all participants demonstrated a decrease in risky responses after punishment trials (Leland and Paulus, 2005).

To investigate the time pressure effect, Farashahi et al. (2018) studied how participants determine the amount of time in collecting sensory information to make a perceptual decision when the reward for correct choice decreases over time. They found that sensory integration over time is imperfect and deteriorates under time pressure. Furthermore, the study demonstrated that when the cost of time is a factor, decision processes are influenced by limitations in sensory integration. Urgency is related to collapsing boundaries model, which explains urging individuals to make choices with less evidence, potentially leading to suboptimal outcomes (Bowman et al., 2012; Drugowitsch et al., 2012; Hawkins et al., 2015). Based on a collapsing boundaries model, a theoretical study suggested that the presence of an opt-out option might prevent rushing to judgment and making suboptimal choices as the escape option would effectively preempt the collapse of decision boundaries (Vargas and Lauwereyns, 2021).

### Rationale of the present study

This study investigated the impact of an opt-out option as a third decision alternative on decision-making strategies under varying levels of pressure, including penalties, task difficulty, and time pressure. Building upon previous research (Farashahi et al., 2018; Miletic and van Maanen, 2019), we adapted a time–pressure decision-making task including an additional response option with the ability to skip a trial without incurring a penalty. This modification aimed to explore how the availability of an escape mechanism would influence decision-making behavior.

We hypothesized that participants would strategically utilize the opt-out option in trials in which they experienced uncertainty, in order to achieve reduced error rates and improved overall performance. In Experiment 1, participants were required to select the square flashing more frequently from two simultaneously presented stimuli. Given the relatively low penalty in Experiment 1, we anticipated that increasing the punishment in Experiment 2 would motivate participants to opt out more frequently. As a result, we anticipated that participants would be more likely to opt out of decisions, particularly in challenging situations. In Experiment 3, we examined the influence of the opt-out option on decision making under varying standards of difficulty and time pressure. Participants were required to identify the dominant color in a patch of red and green dots. We expected that participants would strategically utilize the opt-out option more often when stimulus discrimination was more difficult, and when deadlines were shorter, in efforts to improve their decision performance.

## Methods

### Frequency judgment: experiment 1 (low cost) and experiment 2 (high cost)

#### Participants

In Experiment 1 (low cost), there were 24 participants with a mean age of 24 ± 4.5 years old, recruited via Kyushu University; 17 were females, and only one was left-handed. In Experiment 2 (high cost), there were 26 participants with a mean age of 24 ± 3.5 years old, recruited via Kyushu University; 17 were males, and only three were left-handed. The study was conducted as an exploratory study, setting the sample size at 24 for each experiment, before closing the signup. In Experiment 2 (high cost), an extra two students had already signed up before the portal was closed.

The procedures for this study were conducted in strict accordance with the ethical guidelines outlined in the Declaration of Helsinki. The study was approved by the Human Ethics Committee of the Faculty of Arts and Science, Kyushu University (approval number 202205). All participants gave prior written consent and were requested to fill out participant questionnaire before and after experiment. Each participant received either course credit or monetary compensation of 1,000 yen for their participation. An experimental session lasted around 1 h. All participants’ data were included in the data analysis.

#### Apparatus

A computer with 23.8-inch screen was used to present the stimuli and a wireless keyboard was used for collecting responses from the participants. All stimulus and recordings were controlled through code written in Psychopy (version 1.90.3) (Peirce, 2007, 2009). The distance between the display and the participant was approximately 75 cm.

#### Procedure

For both Experiment 1 (low cost) and Experiment 2 (high cost), the experimental session comprised of two perceptual decision-making tasks, Condition 1 (basic) and Condition 2 (with skip). To prevent an order effect, we counterbalanced the order of Condition 1 and Condition 2. Prior to starting each task, participants received both verbal and written instructions for each task to ensure comprehension.

##### Condition 1 (basic)

**Figure 1:**
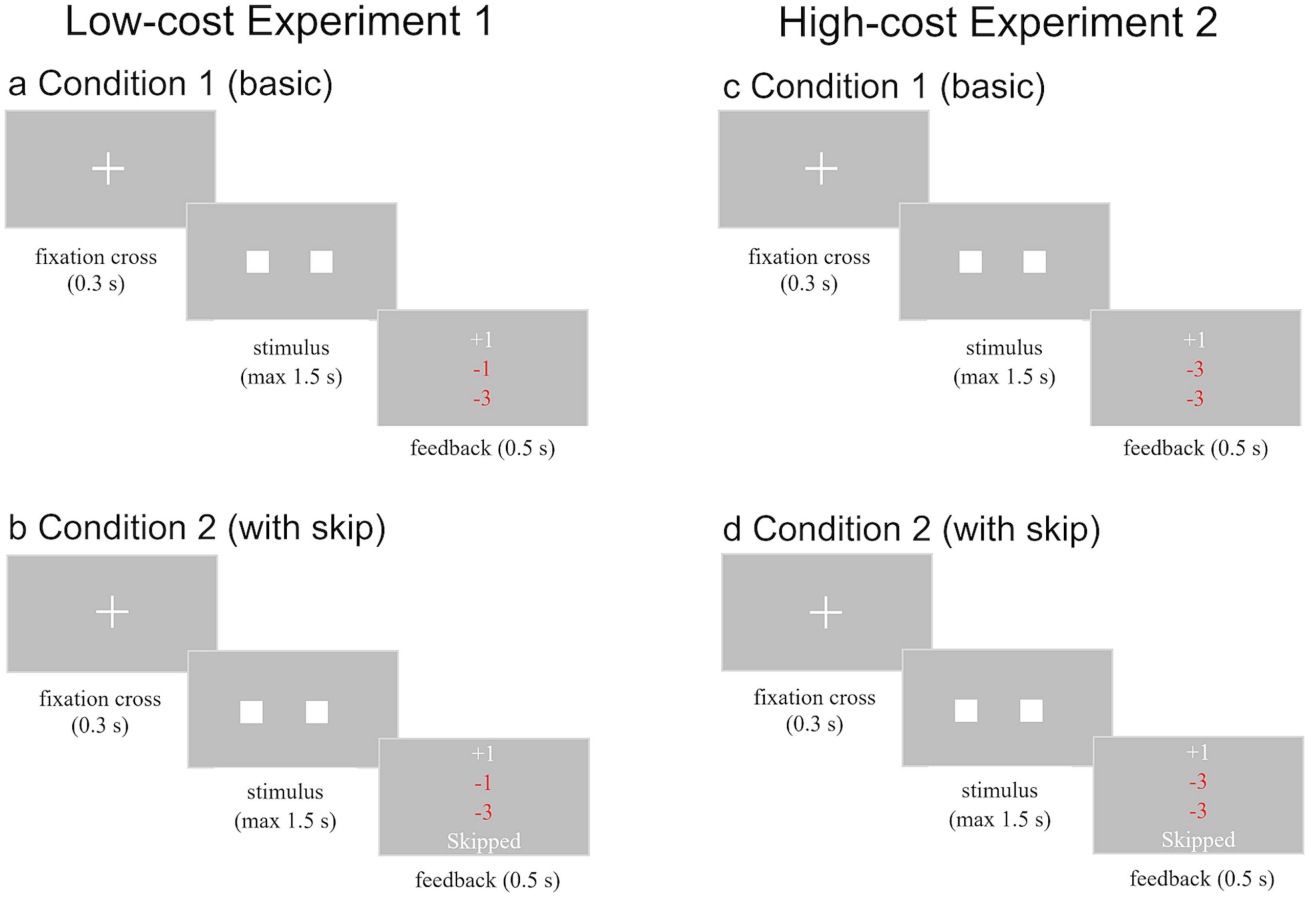
Schematic of experimental paradigms for Frequency Judgment: **(a)** represents Condition 1 (basic) in Experiment 1 (low cost). A brief fixation cross precedes the presentation of two flashing squares. Participants respond by indicating which square flashed more frequently using left or right button presses. Feedback is provided after each trial. **(b)** shows Condition 2 (with skip) in Experiment 1. Similar to Condition 1, but participants can also choose to skip the trial by pressing the space bar. Feedback includes options for correct, incorrect, too late, or skipped responses. **(c,d)** represent Condition 1 (basic) and Condition 2 (with skip) in Experiment 2 (high cost). The penalty for an incorrect answer was increased from 1 to 3 points in Experiment 2.

In the first perceptual decision-making task, participants were required to select the square with the higher flicker frequency from two simultaneously presented stimuli (Figure 1a). Participants were instructed to respond as fast as possible while minimizing errors. Each trial began with a fixation cross displayed for 0.3 s, followed by the presentation of two squares positioned to the right and left of the fixation cross. Both squares exhibited a constant flicker frequency throughout the trial. Every square flashed for 0.050 s, followed by disappearance for 0.0667 s (corresponding to 3 and 4 frames on a 60 Hz computer monitor). The probability of the correct answer square flashing was p-correct = 0.7, while the incorrect square flashed with a probability of p-incorrect = 0.3. These frequency parameters aligned with previous research (Miletic and van Maanen, 2019). Participants indicated their choice by pressing the right arrow key for the right square or the left arrow key for the left square, within a 1.5-s deadline.

In Experiment 1 (low cost), the scoring system rewarded correct answers with one point, penalized incorrect answers by one point, and deducted three points for responses that missed the deadline. In Experiment 2 (high cost), the scoring system penalized incorrect answers by three points but remained the same for correct answers and for responses that missed the deadline. Thus, the only difference between Experiment 1 and Experiment 2 was that the cost for an incorrect answer was higher in Experiment 2 (three points; versus only one point in Experiment 1). The task concluded upon reaching a total score of 120 points or after completing 300 trials. A training block of ten trials was administered prior to the formal task.

Following each response, participants received feedback on their performance for that trial. As depicted in Figure 1a, in Experiment 1 (low cost), feedback consisted of a point value: “+1” in white for correct answers, “–1” in red for incorrect answers, and “–3” in red for responses exceeding the 1.5-s deadline. As depicted in Figure 1c, in Experiment 2 (high cost), the only change was for incorrect answers, with “–3” in red. Points were accumulated across trials with total score feedback provided every ten trials. Participants advanced to the next trial by pressing the space bar upon receiving total score feedback.

##### Condition 2 (with skip)

The second perceptual decision-making task was similar to Condition 1, except that in this task, participants were provided with an additional “skip” option for responding. The sequence and stimuli of this task were identical to Condition 1, as depicted in Figure 1 (see panels b and d). The task commenced with a 0.3-s fixation cross, followed by the presentation of two squares from which participants selected the one flashing more frequently. Feedback was provided after each response. Contrary to Condition 1, participants could respond using not only arrow keys but also the space bar to skip the trial. The point allocation system mirrored Condition 1; no points were awarded or deducted if participants skipped the trial (i.e., pressing the space bar) before 1.5 s. Similar to Condition 1, Condition 2 (with skip) concluded upon reaching a total score of 120 points or after completing 300 trials. A training block of ten trials preceded the formal task. Upon completing all experimental tasks, participants were administered a questionnaire related to their performance.

### Color judgment: experiment 3

#### Participants

Thirty-six participants with a mean age of 26 ± 4.2 years were recruited via Kyushu University. Nineteen were male, and only three were left-handed. The sample size was set by a power analysis using the software program G*Power. Our goal was to obtain 0.95 power to detect a medium effect size of 0.2 at the standard 0.05 alpha error probability. Prior to the experiment, all participants provided written informed consent and completed a participant questionnaire. The experiment involved two visits, with participants receiving a monetary incentive of 2,000 yen for their participation in the second visit. Data from all participants were included in the data analysis.

##### Apparatus

A 23.8-inch computer monitor was employed to present stimuli, while a wireless keyboard was utilized to record participant responses. All stimuli and recordings were managed through code written in Psychopy (version 2023.1.2) and using JASP software for data analysis (Love et al., 2019). The viewing distance between the participant and the display was approximately 75 cm.

#### Procedure

The experiment comprised one pretest task and two primary tasks, all of which involved perceptual decision-making. To prevent an order effect, we counterbalanced the order of Condition 1 and Condition 2. The tasks were explained verbally and separately, with written instructions, prior to starting each task to ensure participants’ understanding.

##### Pre-test task

In the pre-test task, participants were required to identify the dominant color within a patch of red and green dots. Each trial commenced with a white fixation cross displayed for 1.5 s, followed by the presentation of two fixed-colored circles (150, 150 pixels). A white arc (0.6, 0.6 pixels) appeared on the screen for 1.0 s. The arc represented the available time. The fixation cross then transitioned to a display of green and red dots (100 dots in total, each one 10 pixels) randomly distributed across the screen, while the arc began to decrease. Participants were instructed to indicate their response by pressing the right arrow key for red or the left arrow key for green. Upon submitting their response, participants received feedback on their answer for 1.0 s. Feedback indicated the points earned or lost for each trial: “correct +1” in white denoted a correct answer, “incorrect–2” in red indicated an incorrect response, and “–2” in white appeared when participants failed to respond within the allotted time. Points were accumulated through all trials; with total score feedback presented every 18 trials. Upon receiving total score feedback, participants pressed the space bar to continue the remaining trials in the task. The pre-test task comprised 324 trials divided into 18 blocks of 18 trials each. Three levels of difficulty (dot ratio) were incorporated: 60:40, 57:43, and 54:46. Each block included six trials for each difficulty level. Additionally, three different deadlines (time pressure) were implemented: 1.5 s, 2.0 s, and 2.5 s. Difficulties and deadlines were randomly assigned across trials. The task concluded after the presentation of 324 trials.

##### Condition 1 (basic)

**Figure 2:**
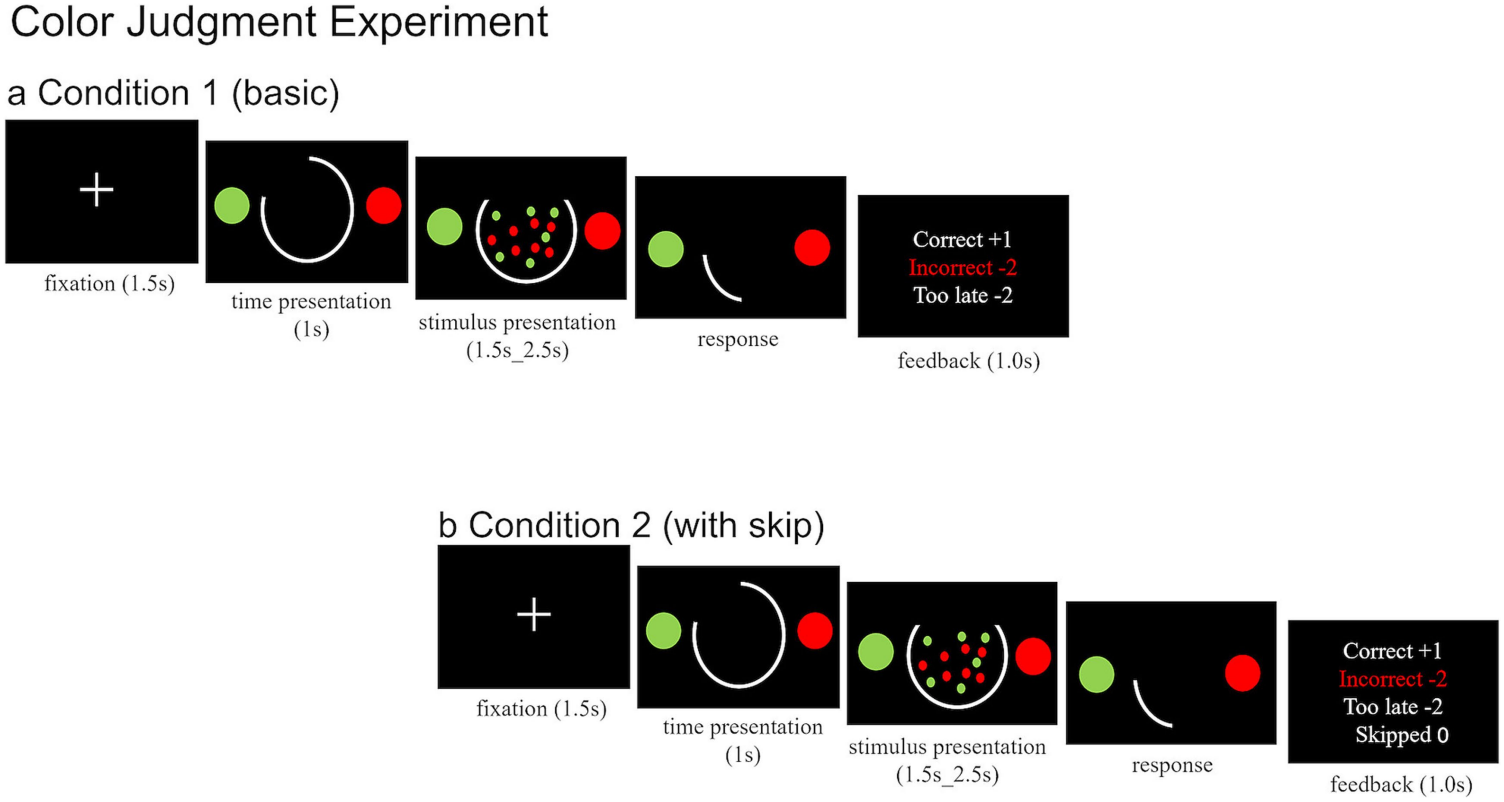
Schematic of experimental paradigms of Color Judgment: **(a)** represents Condition 1 (basic). Short fixation cross is presented, followed by a time indicator and two circles. Subsequently, a constant distribution of red and green dots appears. Participants determine the dominant color by pressing the left or right button. Feedback is provided after each trial. **(b)** Condition 2 is similar to Condition 1, but participants can choose the dominant color or skip the trial using the space bar. Feedback includes options for correct, incorrect, too late, or skipped responses.

In Condition 1, participants were required to identify the dominant color in a patch of red and green dots (Figure 2a). All trials started with a white fixation cross displayed for 1.5 s, then two-colored targets and a white arc displayed on the screen for 1.0 s. The arc represented the trial deadline. The fixation cross was replaced by green and red dots randomly distributed across the middle of the screen, while the arc began to decrease. Participants were instructed to indicate their response by pressing the right arrow key for red or the left arrow key for green (Farashahi et al., 2018). Subsequently, participants received feedback on their response for 1.0 s. As illustrated in Figure 2a, feedback displayed the points earned or lost for each trial: “Correct +1” in white indicated a correct response, “Incorrect–2” in red denoted an incorrect response, and “Too late–2” in white appeared when participants failed to respond within the allotted time. Points were accumulated across all trials, with feedback on the total score provided every 18 trials. Upon receiving total score feedback, participants pressed the space bar to continue the remaining trials. Condition 1 comprised 240 trials divided into 15 blocks of 16 trials each, representing two difficulty levels and two deadlines (2 × 2). For each subject, we set the dot ratio and deadline that produced the pre-test correct rate nearest to 80% as well as a more difficult dot ratio and shorter deadline. Each block included eight trials for each difficulty. The conditions were randomly assigned across trials. The task concluded upon reaching a total score of 100 points or after completing 240 trials.

##### Condition 2 (with skip)

Condition 2 was similar to Condition 1, with the same difficulty levels and deadlines, requiring participants to identify the dominant color within a patch of red and green dots (Figure 2b). However, in Condition 2, participants had an additional response option to skip the trial. After each response, participants received feedback. As illustrated in Figure 2b, feedback indicated the points earned or lost for each trial: “Correct +1” in white denoted a correct response, “Incorrect–2” in red indicated an incorrect response, “Too late–2” in white appeared when participants failed to respond on time, and no points were awarded or deducted for skipped trials (i.e., pressing the space bar). Points were accumulated across all trials, with feedback on the total score provided every 18 trials. Upon receiving total score feedback, participants pressed the space bar to continue the remaining trials in the task. Similar to Condition 1, the task concluded upon reaching a total score of 100 points or after completing 240 trials. Following completion of all the tasks, participants were administered a questionnaire related to the experiment.

## Results

In all experiments, in both conditions with and without a skip option, accuracy was calculated as the number of correct trials divided by the total number of correct, incorrect, and late trials. The accuracy was not measured on the basis of points earned.

We calculated the fraction of skipped trials as the number of skipped trials divided by the total number of correct, incorrect, and late trials.

### Experiment 1 (low cost)

**Figure 3:**
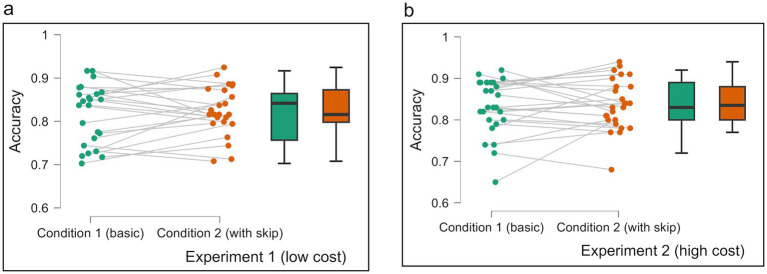
**(a,b)** Average ratings in each condition in Experiment 1 (low cost) and Experiment 2 (high cost). Each panel shows data of accuracy in Condition 1 (basic) and Condition 2 (with skip) for both experiments. The green bar represents the result of Condition 1, and the orange bar represents the result of Condition 2. The average of Condition 2 is without calculating skip trials. Error bars represent 95% CI to the upper and lower ends. The solid horizontal bar inside the colored rectangles represents the median. The upper and lower ends of the colored rectangles represent the interquartile range (IQR), specifically the 25th and 75th percentiles. The whiskers extending from the boxes typically represent the range of the data, often within 1.5 times the IQR from the box edges, and dots represent individual data points.

Experiment 1 (low cost) investigated the relationship between time pressure and utilization of an opt-out option in decision-making (Figure 3a). We applied two conditions, a basic condition requiring a forced choice (Condition 1) and a condition with an added option to skip the decision without penalty (Condition 2). Participants were allotted a 1.5-s response window for each trial, incurring a penalty for delayed decisions. In the results, there was no significant difference in accuracy between Condition 1 (*M* = 0.82, *SD* = 0.07) and Condition 2 [(*M* = 0.82, *SD* = 0.06), *t* (23) = –0.7, *p* = 0.482]. The average performance was very high, with only a small amount of skipping in Condition 2, presumably because the participants were able to achieve a high accuracy without any significant risk for erroneous answers. Therefore, in Experiment 2 we raised the risk for erroneous answers by increasing the punishment from–1 to–3 to examine whether the participants would make more use of the skipping option.

### Experiment 2 (high cost)

In Experiment 2, the average performance was similar between the basic and with-skip conditions; a paired sample T-test indicated there was no significant difference in accuracy between Condition 1 (*M* = 0.83, *SD* = 0.07) and Condition 2 [(*M* = 0.84, *SD* = 0.06), *t* (25) = –0.56, *p* = 0.566] (Figure 3b). Independent samples T-test showed no significant difference in accuracy between Experiment 1 (*M* = 0.825, *SD* = 0.06) and Experiment 2 (*M* = 0.840, *SD* = 0.06) in Condition 2, *t* (48) = –0.708, *p* = 0.482.

**Figure 4:**
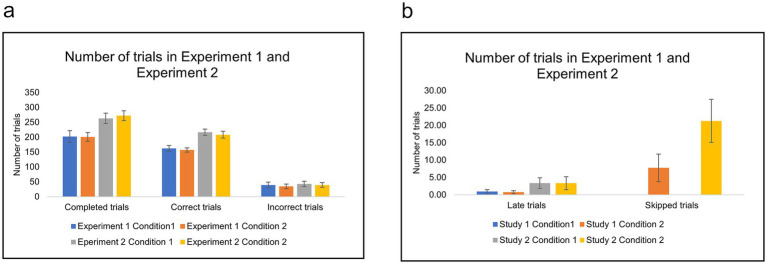
**(a,b)** The number of trials in Condition 1 (basic) and Condition 2 (with skip) of Experiment 1 (low cost) and Experiment 2 (high cost). Error bars indicate 95% confidence intervals to the upper and lower ends.

A two-factor repeated measures ANOVA indicated that correct trials increased significantly in Experiment 2 compared to Experiment 1, *F* (1, 23) = 69.4, MSE = 62475.010, ${\eta^{2}}_{p}$ = 0.751, *p* < 0.001. *Post-hoc* pairwise comparisons showed that correct trials increased in Condition 1 of Experiment 2 (*M* = 214.458, *SD* = 27.5) compared with Condition 1 of Experiment 1 (*M* = 162.125, *SD* = 25.2) In addition, correct trials increased in Condition 2 of Experiment 2 (*M* = 206.667, *SD* = 30.5) compared with Condition 2 of Experiment 1 (*M* = 156.958, *SD* = 18.4). These results indicate that raising punishment resulted in increased correct trials. We note that the number of completed trials in Experiment 2 was significantly higher than in Experiment 1; participants needed more trials to earn the required number of points to finish the task (Figure 4a). In addition, the number of late trials increased significantly in Experiment 2 compared with Experiment 1 in all conditions (Figure 4b).

**Figure 5:**
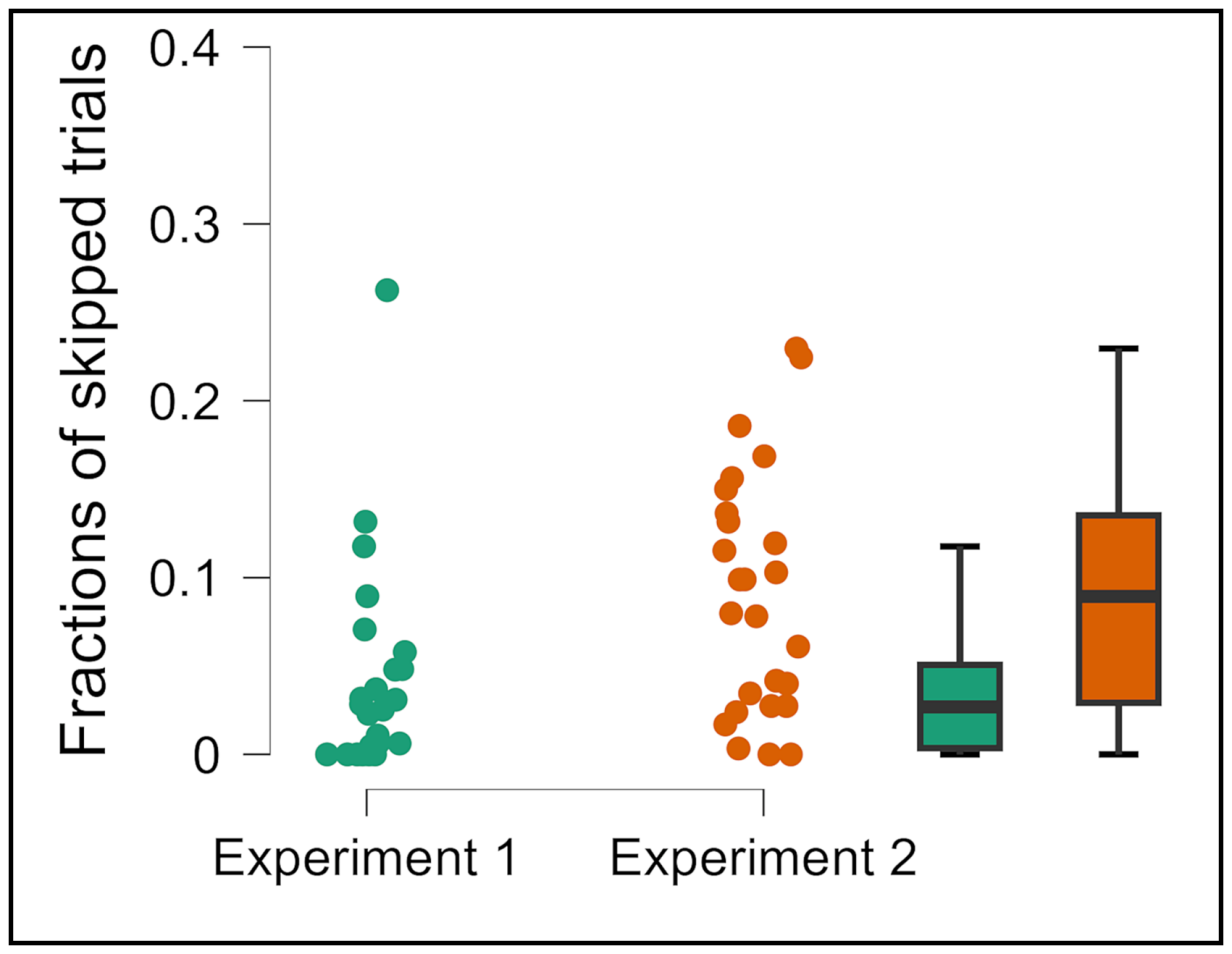
Fractions of skipped trials in Condition 2 of Experiment 1 and Experiment 2. Error bars represent 95% CI to the upper and lower ends. The solid horizontal bar inside the colored rectangles represents the median. The upper and lower ends of the colored rectangles represent the interquartile range (IQR), specifically the 25th and 75th percentiles. The whiskers extending from the boxes typically represent the range of the data, often within 1.5 times the IQR from the box edges, and dots represent individual data points.

Moreover, participants skipped proportionally more in Experiment 2 (high cost) (*M* = 0.090, *SD* = 0.068) compared with Experiment 1 (low cost) (*M* = 0.043, *SD* = 0.060), *t* (48) = −2.608, *p* = 0.012 (Figure 5). However, this did not translate into higher accuracy (Figure 3).

**Figure 6:**
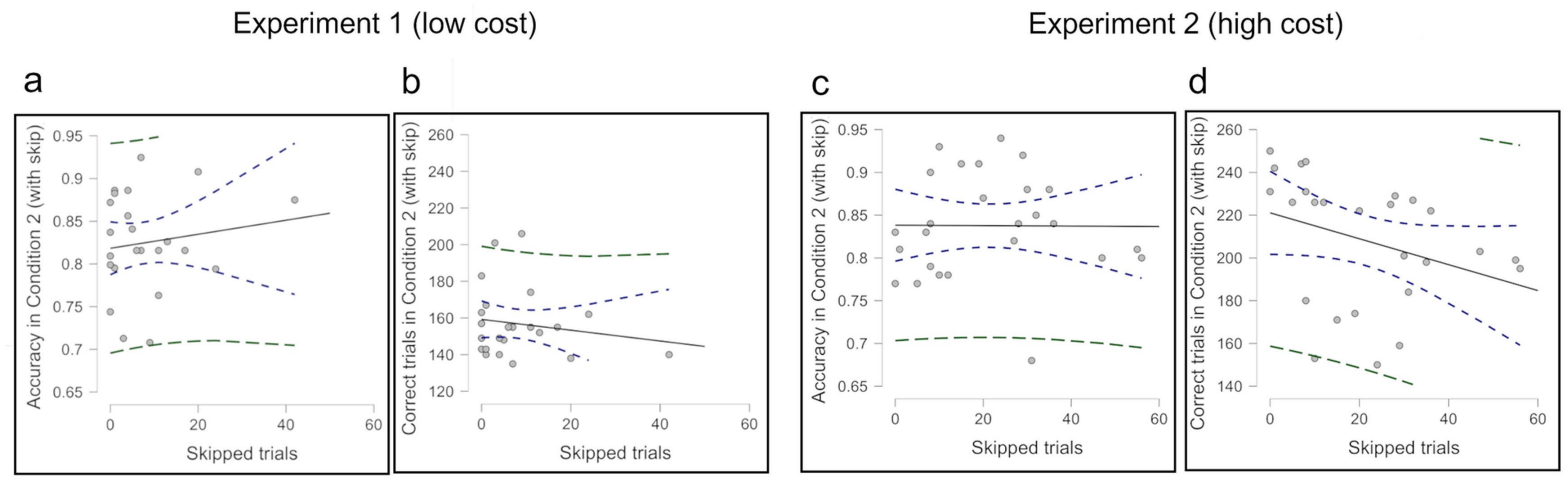
**(a)** Correlation between skipped trials and overall accuracy in Condition 2 (excluding skipped trials) in Experiment 1. **(b)** Correlation of skipped trials versus the number of correct trials in Condition 2 in Experiment 1. **(c)** Correlation between skipped trials and overall accuracy in Condition 2 (excluding skipped trials) in Experiment 2. **(d)** Correlation of skipped trials versus the number of correct trials in Condition 2 in Experiment 2.

We examined the correlation between skipped trials and both accuracy and correct trials in Experiment 1 (low cost) (Figures 6a,b) and Experiment 2 (high cost) (Figures 6c,d). Opt-out choice affected correct trials negatively, but no significant correlations were found; Experiment 1 *r* (22) = –0.159, *p* = 0.457; Experiment 2 *r* (24) = −0.329, *p* = 0.101. There were also no significant correlations with accuracy; Experiment 1 *r* (22) = 0.145, *p* = 0.500; Experiment 2 *r* (24) = −0.007, *p* = 0.974.

**Figure 7:**
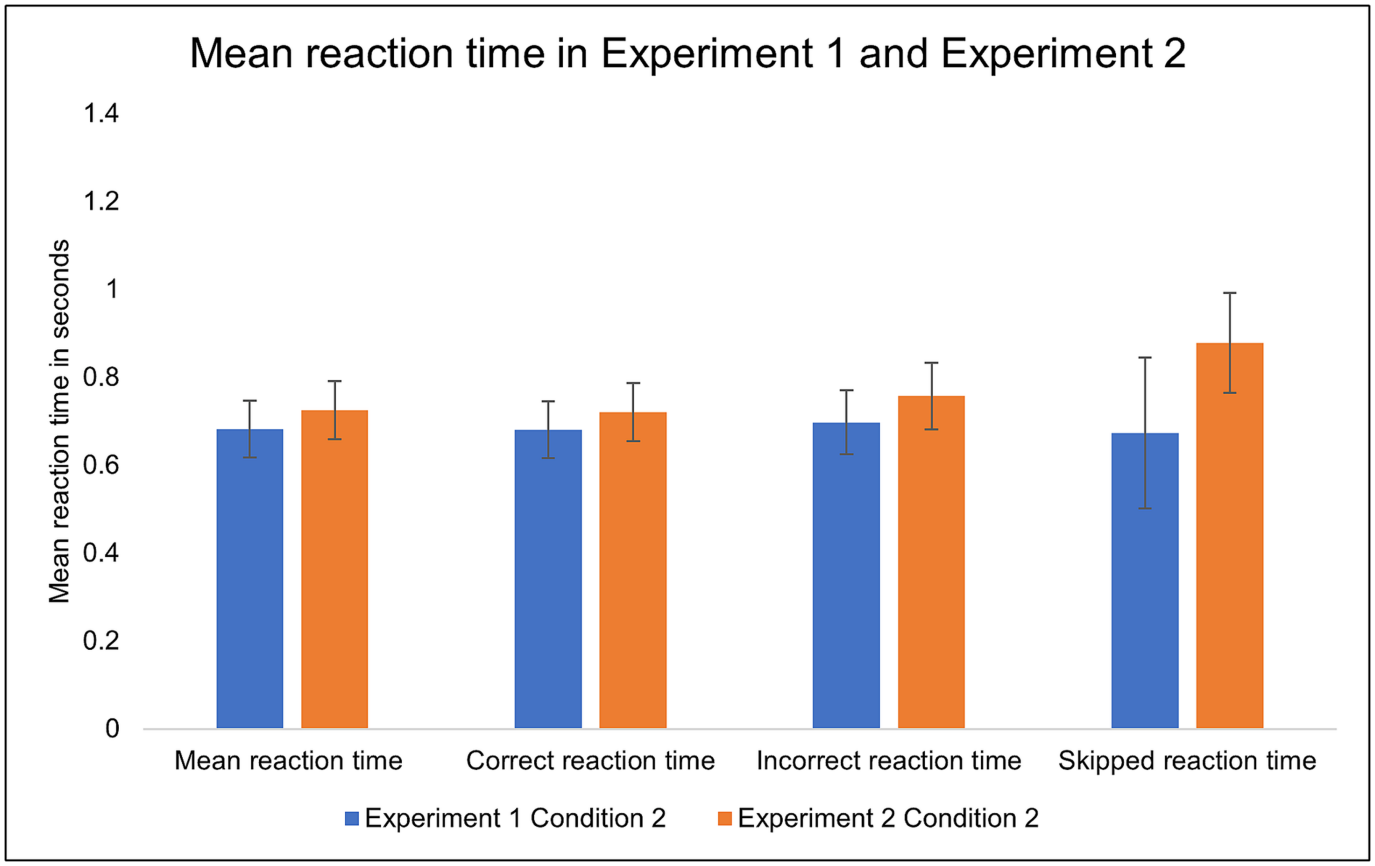
The mean reaction times in Condition 2 (with skip) of Experiment 1 (low cost) and Experiment 2 (high cost). Error bars indicate 95% confidence intervals to the upper and lower ends.

To examine the skipping behavior further, we analyzed the reaction times. The reaction time when skipping tended to be marginally closer to the deadline in Experiment 2 (*M* = 0.88, *SD* = 0.29) than in Experiment 1 (*M* = 0.67, *SD* = 0.43), *t* (48) = −1.98, *p* = 0.052 (Figure 7).

### Experiment 3

**Figure 8:**
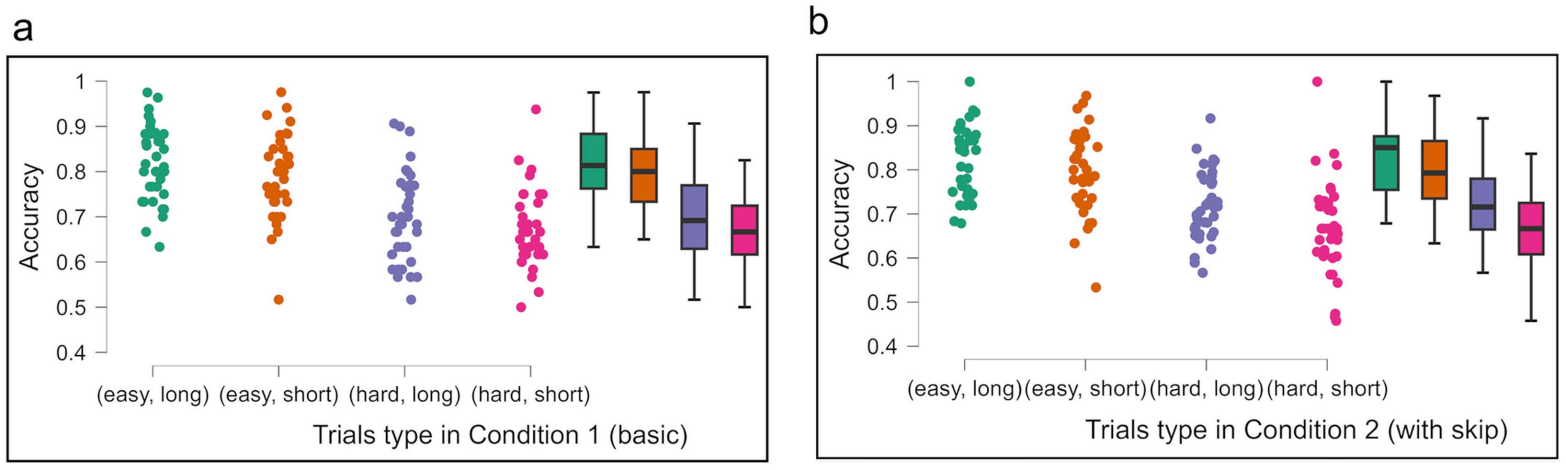
Average accuracy across task difficulty and time pressure conditions in Experiment 3. Each panel shows data for (easy, long), (easy, short), (hard, long) and (hard, short) in Condition 1 **(a)** and Condition 2 **(b)**. The average of Condition 2 is without calculating skip trials. Error bars represent 95% CI to the upper and lower ends. The solid horizontal bar inside the colored rectangles represents the median. The upper and lower ends of the colored rectangles represent the interquartile range (IQR), specifically the 25th and 75th percentiles. The whiskers extending from the boxes typically represent the range of the data, often within 1.5 times the IQR from the box edges, and dots represent individual data points.

To further explore the factors influencing opt-out behavior, Experiment 3 employed a color judgment task with varying levels of difficulty and time pressure. Increased perceptual difficulty and shorter deadlines caused a decline in accuracy (Figures 8a,b). A three-way repeated measures ANOVA produced a significant decline in correct rates across difficulty levels, *F* (1, 35) = 335.982, MSE = 4363.337, ${\eta^{2}}_{p}$ = 0.906, *p* < 0.001, and time pressure levels *F* (1,35) = 18.530, MSE = 327.253, ${\eta^{2}}_{p}$ = 0.346, *p* < 0.001. However, the analysis indicated no significant difference in accuracy between the basic and skip conditions, *F* (1, 35) = 0.55, MSE = 0.003, ${\eta^{2}}_{p}$ = 0.016, *p* = 0.462 (Figures 8a,b).

**Figure 9:**
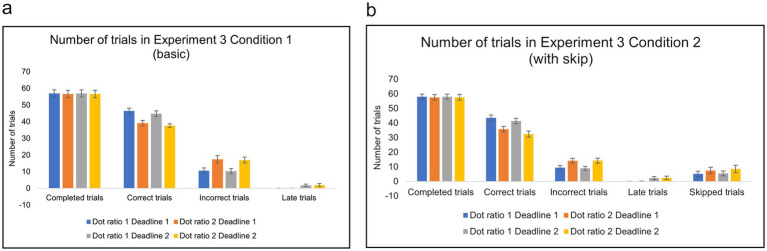
**(a,b)** The number of trials in Condition 1 (basic) and Condition 2 (with skip) of Experiment 3. Error bars indicate 95% confidence intervals to the upper and lower ends.

**Figure 10:**
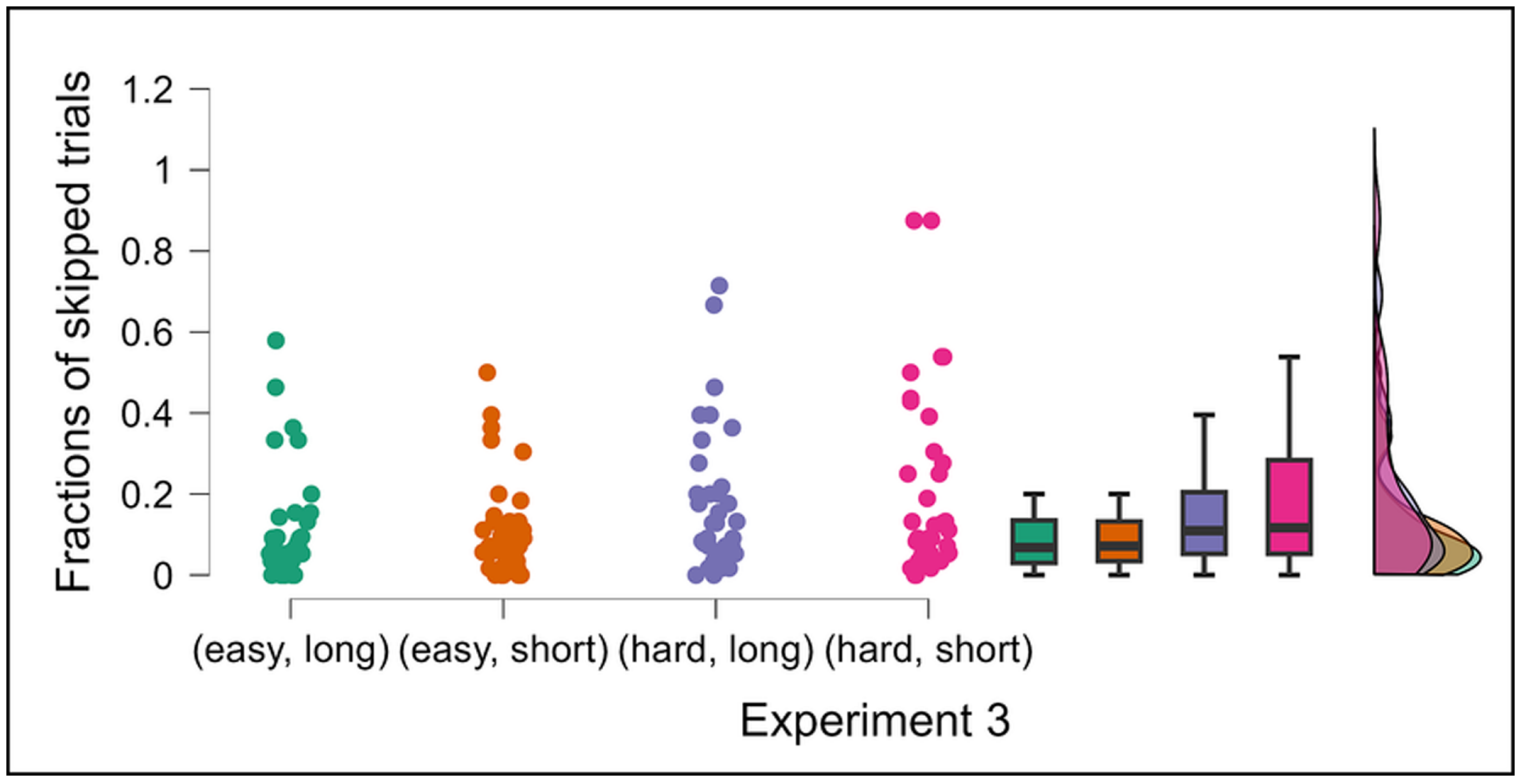
Fractions of skipped trials per type of trials in Condition 2 of Experiment 3. Data are presented for four experimental conditions: (easy, long), (easy, short), (hard, long) and (hard, short) in Condition 2. Error bars indicate 95% confidence intervals to the upper and lower ends. The solid horizontal bar inside the colored rectangles represents the median. The upper and lower ends of the colored rectangles represent the interquartile range (IQR), specifically the 25th and 75th percentiles. The whiskers extending from the boxes typically represent the range of the data, often within 1.5 times the IQR from the box edges, and dots represent individual data points.

The numbers of trials are presented in Figure 9, with fractions of skipping in Figure 10. A two-factor repeated measures ANOVA analysis on the number of correct trials revealed that participants with an option to skip (Condition 2) achieved significantly fewer correct trials across all difficulty and time pressure levels *F* (1, 35) = 20.674, MSE = 1001.281, ${\eta^{2}}_{p}$ = 0.371, *p* < 0.001. *Post-hoc* pairwise comparisons indicated that correct trials decreased significantly in hard/short level in Condition 2 (*M* = 32.42, *SD* = 6.49) compared with Condition 1 (*M* = 37.67, *SD* = 3.46) *p* < 0.001, hard/long level in Condition 2 (*M* = 35.81, *SD* = 5.89) compared Condition 1 (*M* = 39.14, *SD* = 4.82), *p* = 0.031, easy/short level in Condition 2 (*M* = 41.42, *SD* = 5.72) compared with Condition 1 (*M* = 44.83, *SD* = 5.36) *p* = 0.024.

With respect to skipping, in Condition 2, a two-factor repeated measures ANOVA showed that opt-out fraction significantly increased under more difficult conditions, *F* (1, 35) = 29.397, MSE = 0.216, ${\eta^{2}}_{p}$ = 0.456, *p* < 0.001. *Post-hoc* pairwise comparisons showed that skipped fractions increased significantly in hard/long level (*M* = 0.172, *SD* = 0.178) compared with easy/long level (*M* = 0.110, *SD* = 0.139) *p* = 0.023, hard/short level (*M* = 0.208, *SD* = 0.228) compared with easy/short level (*M* = 0.115, *SD* = 0.122) *p* < 0.001. However, increased opt-out rates did not lead to improved accuracy (Figure 8).

**Figure 11:**
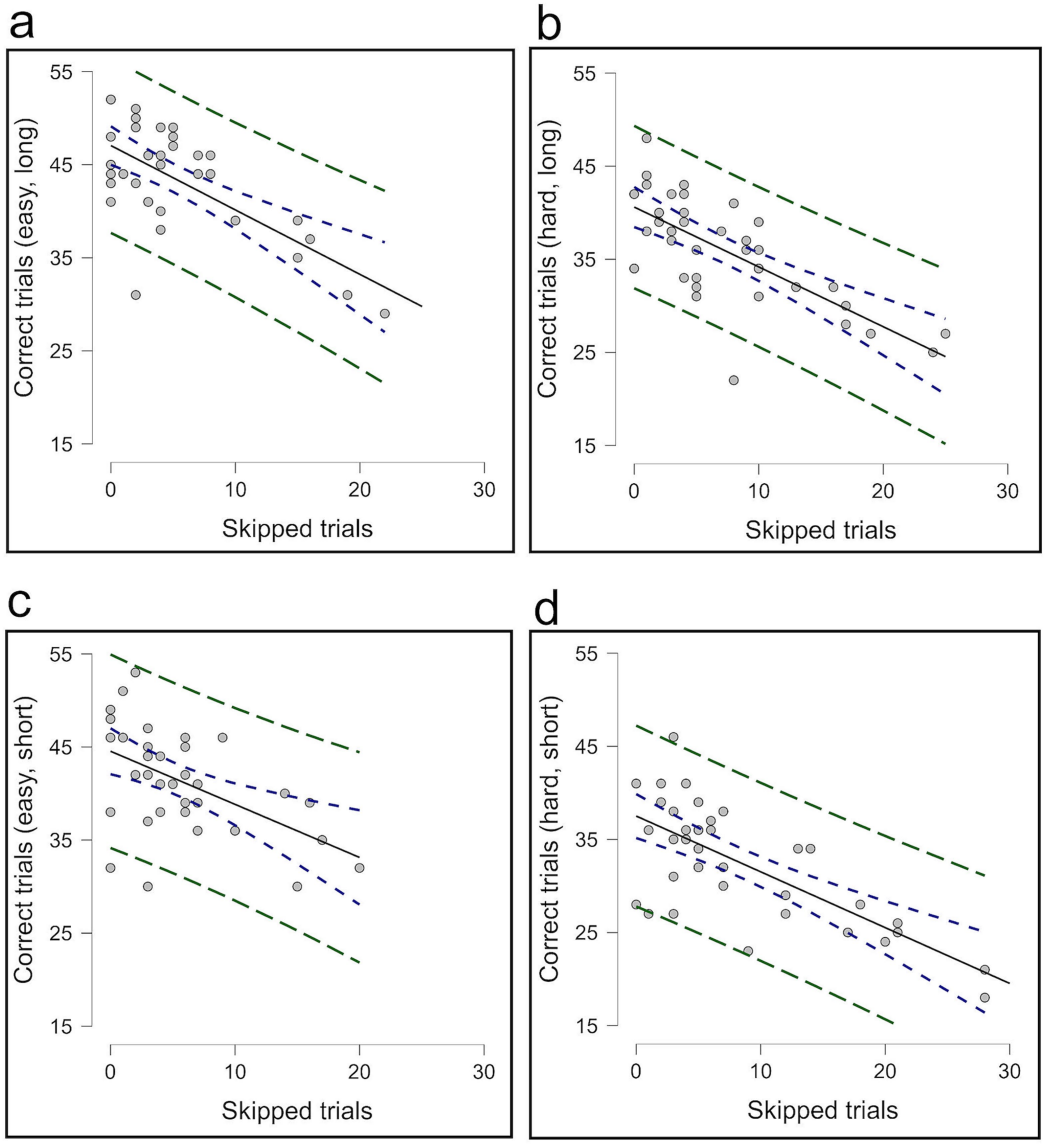
The correlation between skipped trials and correct trials in Condition 2. Panels **(a,b)** present the correlation for easy and hard trials under long conditions, while panels **(c,d)** show the correlation for easy and hard trials under short conditions.

**Figure 12:**
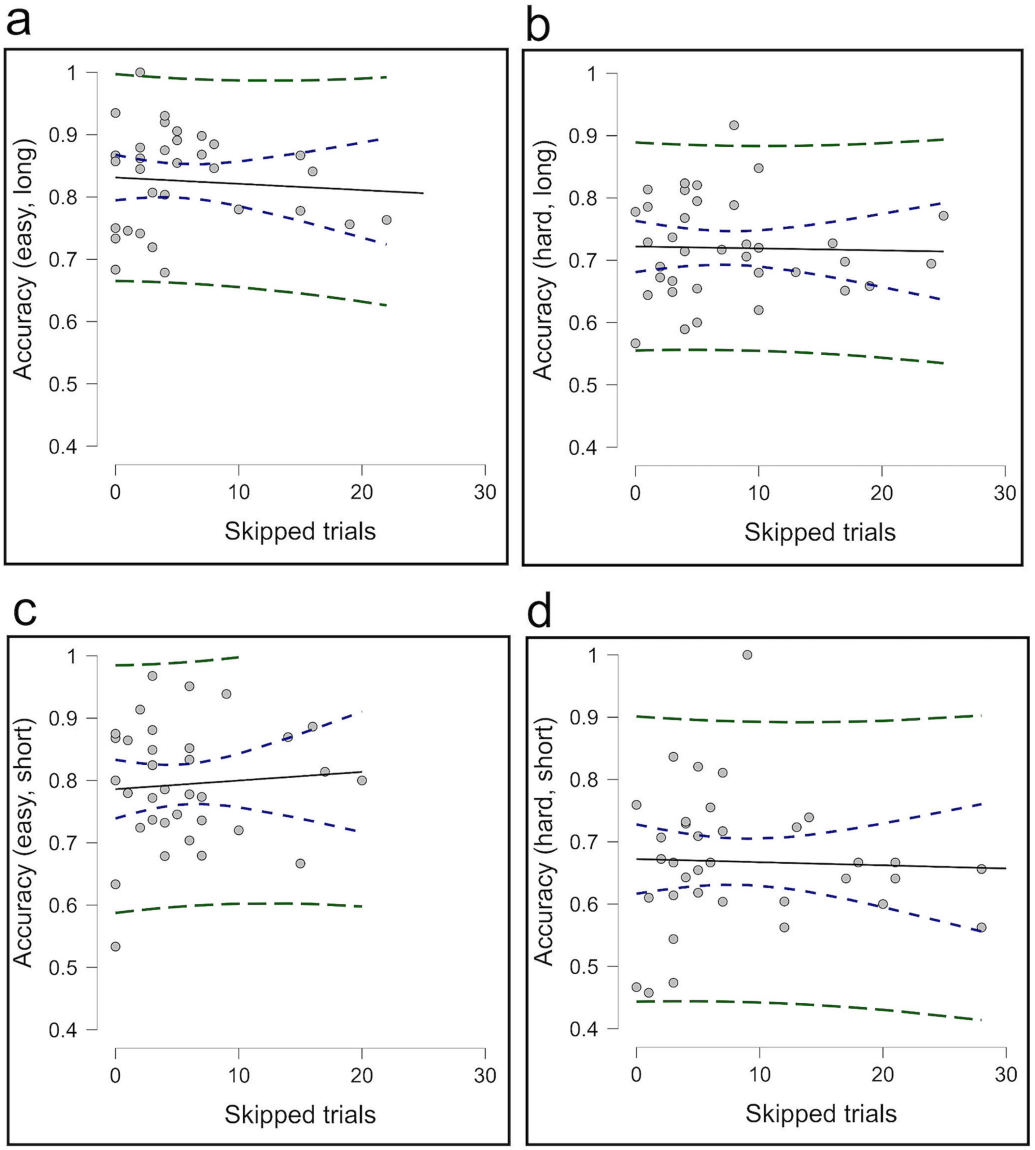
The correlation between skipped trials and accuracy in Condition 2. Panels **(a,b)** present the correlation for easy and hard trials under long conditions, while panels **(c,d)** show the correlation for easy and hard trials under short conditions. The average of Condition 2 is without calculating skip trials.

In Experiment 3, correlational analyses indicated that participants who opted out more frequently tended to make fewer correct choices across all levels of difficulty and time pressure in Condition 2; easy/long level [*r* (34) = −0.664, *p* < 0.001]; hard/long level [*r* (34) = −0.718, *p* < 0.001]; easy/short level [*r* (34) = −0.517, *p* = 0.001]; and hard/short level [*r* (36) = −0.710, *p* < 0.001] (Figure 11). However, no significant correlation was observed between opt-out use and overall accuracy; easy/long level [*r* (34) = −0.073, *p* = 0.672]; hard/long level [*r* (34) = −0.027, *p* = 0.878]; easy/short level [*r* (34) = 0.076, *p* = 0.658]; and hard/short level [*r* (36) = −0.036, *p* = 0.835] (Figure 12), suggesting that participants who opted out more frequently made fewer correct choices, which did not help raise overall performance.

**Figure 13:**
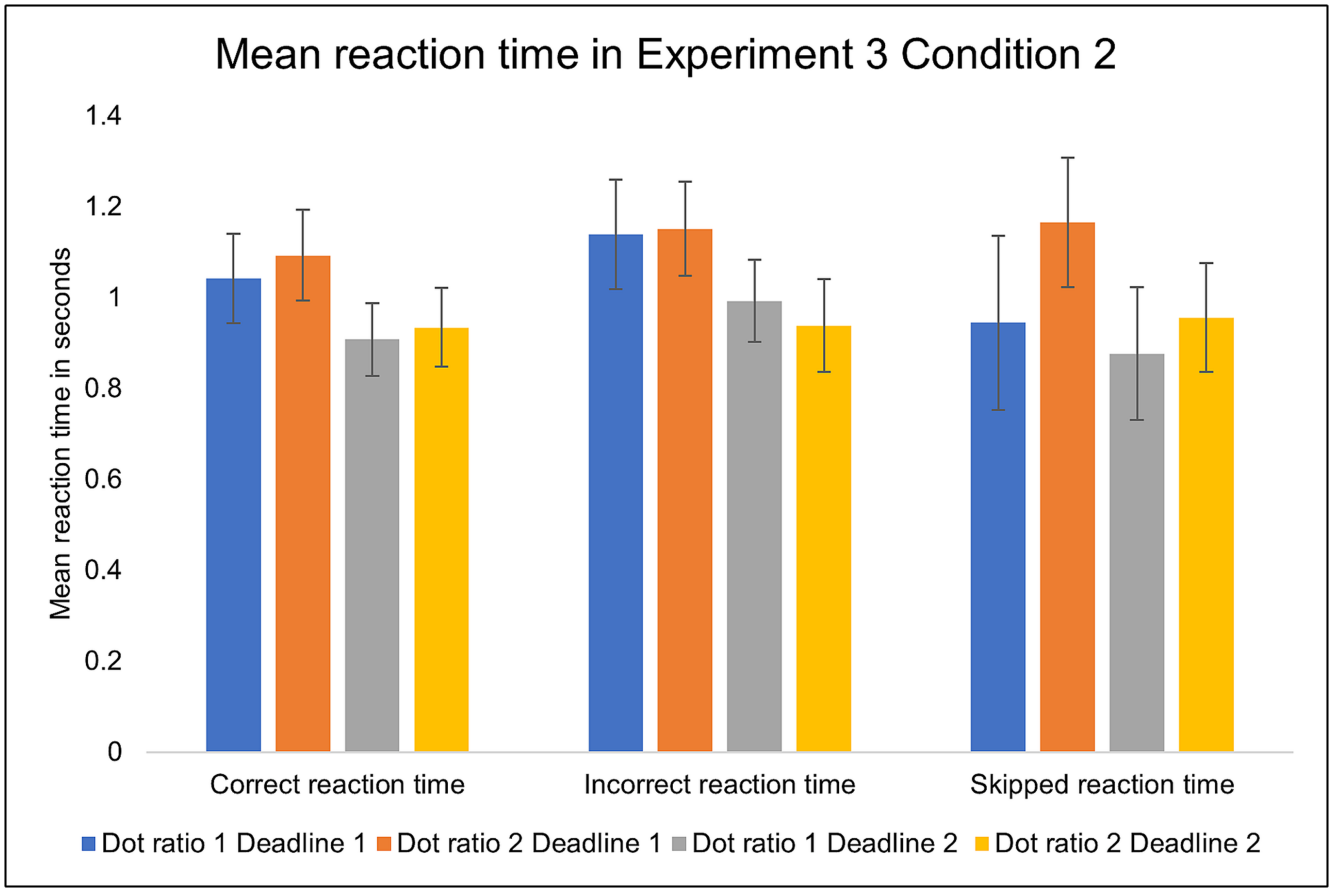
The mean reaction times in Condition 2 (with skip) of Experiment 3. Error bars indicate 95% confidence intervals to the upper and lower ends.

In Experiment 3, the results of reaction time of skipped trials were mixed. The mean reaction time was significantly delayed in the more difficult trials when the deadline was long: Dot ratio 1 (*M* = 0.94, *SD* = 0.59) versus Dot ratio 2 (*M* = 1.16, *SD* = 0.43), *t* (35) = −2.54, *p* = 0.016. For the shorter deadline, there was no significant difference (Figure 13).

## Discussion

Three experiments were conducted to examine how the inclusion of an opt-out option impacts on perceptual decision-making under varying kinds of pressure. We hypothesized that individuals may strategically utilize an opt-out option to reduce errors and improve performance. However, in Experiment 1 (low cost), participants rarely made use of the opt-out option, and it did not affect the decision performance. In Experiment 2 (high cost), the increased risk induced participants to utilize the opt-out option more often, proving that they regarded the escape opportunity as a relevant solution. However, the escape behavior did not improve the decision accuracy. Similarly, in Experiment 3, the participants skipped trials more often on trials with hard-to-discriminate stimuli. However, the reliance on the opt-out option did not lead to improved quality of performance.

It was shown previously that in the absence of deadline, people choose decision criteria that gain the maximum reward, whether monetary or not (Edwards, 1965; Gold and Shadlen, 2002). However, in our study the accuracy was not elevated even when offering the opportunity to opt out of a forced choice.

Our results suggested that the relatively low penalty in Experiment 1 may not have been sufficient to motivate participants to opt-out more frequently. Therefore, we raised the punishment in Experiment 2 and examined the impact of high cost on decision-making. Regarding punishment, it is well established in animal behavioral studies that raising punishment significantly alters the choice for a large, risky reward versus a small, safe reward (Crean et al., 2000; Simon et al., 2009; Blaes et al., 2018). Consistent with Negus (2005), participants tend to choose the safe decision (unpunished behavior). Here we confirmed that increasing the pressure on participants by raising the penalty led to greater escape from the decision (skip button). However, opting out did not allow participants to achieve better performance.

We conducted Experiment 3 to investigate the effect of opt-out under different kinds of pressure conditions such as task difficulty and time pressure (Farashahi et al., 2018; Miletic and van Maanen, 2019). Benson and Beach (1996) created time pressure by requiring a deadline that was one standard deviation below the mean response time. Here, we were able to determine the degree of time pressure required for each participant and the appropriate degree of difficulty and compare them with tasks. The results of Experiment 3 showed that there was no difference in accuracy between the basic and with-skip conditions, in line with the first Experiments 1 and 2. Previous research showed that accuracy decreased significantly at higher difficulty levels (Stone and Kadous, 1997; Dodonova and Dodonov, 2013) and shorter deadlines (Payne et al., 1993; Kocher and Sutter, 2006). Having an opt-out option seemed to give participants confidence as they could opt out at any point. Similarly, Veldwijk et al. (2014) showed that 54% of Dutch Type 2 Diabetes Mellitus patients wanted to participate in discrete choice experiments, related to lifestyle program attributes, when the opt-out option was offered. Moreover, participants chose the opt-out option more often when choice tasks were difficult. Individuals achieved higher accuracy when opting out for more difficult items and leaving the easier items to be calculated for accuracy (Koriat and Goldsmith, 1996; Stankov et al., 2022). Participants in our study tended to opt out of hard trials more often than easy trials. Yet, that did not lead to improve their accuracy. Conversely, there was no clear effect of time pressure on escape choice.

In all three experiments, we discovered that factors like risk and task difficulty tended to nudge participants toward engaging in escape behavior during perceptual decision-making, yet these behaviors did not lead to improvements in accuracy. This suggests that while participants were able to monitor their performance, they ultimately lacked the strategic metacognitive control necessary to effectively utilize the opt-out option.

It is likely that, in the current paradigm, the meta-decision to opt out demanded a level of time and cognitive effort that was not easily compatible with the rapid processing required in fast-paced perceptual decision-making tasks. Here, it is important to note that the opting-out mechanism in our study required subjects to initiate an action (pressing the space bar) under time pressure, in order to avoid being penalized for missing the deadline. For comparison, humans, nonhuman primates, and rats can successfully opt out of perceptual decision-making tasks, even with fast-paced perceptual decisions, when there is no punishment for missing a deadline, and the subjects can simply move on to the next trial by withholding any action in response to the current trial (Kepecs et al., 2008; Kiani and Shadlen, 2009). Put differently, the meta-decision in such circumstances may be characterized as a speeded opting in to the next trial. Future research will be required to clarify under which circumstances metacognitive control can improve perceptual decision-making.

In our experiments, participants seemed unable to assess the efficiency of opting out. Despite no tangible benefit from escape behavior, they tended to opt out more frequently under demanding conditions. These findings suggest that participants were only engaging in first-order monitoring—recognizing that their performance deteriorated under challenging conditions and choosing to skip trials as a form of compensation. However, they did not exhibit second-order monitoring, failing to realize that this countermeasure was, in fact, ineffective and did not improve their overall performance.

## Data Availability

The raw data supporting the conclusions of this article will be made available by the authors, without undue reservation.
